# Mental health status compared among rural-to-urban migrant, urban and rural school-age children in Guangdong Province, China

**DOI:** 10.1186/s12888-019-2356-4

**Published:** 2019-12-03

**Authors:** Ningjing Chen, Yongguang Pei, Xijun Lin, Jun Wang, Xiuqing Bu, Ke Liu

**Affiliations:** 1 School of Nursing, Quanzhou Medical College, Quanzhou, 362000 Fujian Province China; 2 School of Nursing, Sun Yat-sen University, Guangzhou, 510080 Guangdong Province China

**Keywords:** Migrant, Rural, Children, Mental health, Predictors

## Abstract

**Background:**

Previous research has documented mental health status among rural-to-urban migrant children (labeled as “migrant children” henceforth) and urban children. However, the findings remain unclear. In addition, far less attention has been paid to rural children’s psychological outcomes. The purpose of this study was to compare mental health status among migrant, urban and rural school-age children in Guangdong Province, China.

**Methods:**

This was a cross-sectional study involving 372 migrant, 254 urban and 268 rural children selected respectively from 3 private schools, 4 public schools and 2 village schools in Guangdong Province, China. Participants provided their socio-demographic information and completed the Strengths and Difficulties Questionnaire (SDQ) to assess mental health. One-way analyses of variance (ANOVAs) and Bonferroni post hoc test were used to evaluate SDQ scores differences. A multiple linear regression analysis was conducted to measure mental health differences among children after controlling for socio-demographics. Chi-square analyses were used to assess differences in the prevalence of mental health problems among children.

**Results:**

Bonferroni post hoc test showed that migrant and rural children reported significantly higher scores than urban peers in emotional symptoms, hyperactivity/inattention and total difficulties score (*p* < 0.01). In addition, migrant children reported a higher peer problems score compared to urban children (*p* < 0.001). In multiple linear regression analysis, rural and migrant children reported significantly a higher total difficulties score than urban children (*p* = 0.046 and 0.024, respectively). Additionally, female gender, having insurance, seldom communicating with parents, and higher monthly household income were negatively associated with a higher total difficulties score. Conversely, children’s father with secondary education was positively associated with a higher total difficulties score. The prevalence of mental health problems among rural, migrant and urban children were 26.5, 18.8 and 15.0% (χ^2^ = 11.41, *p* = 0.003), respectively.

**Conclusions:**

Rural and migrant children reported poorer mental health than urban children. Female gender, having insurance, seldom communicating with parents, and higher monthly household income were associated with better mental health of children. However, children’s father with secondary education was associated with poorer mental health of children. Given the different effects of socio-demographics, further support might be provided accordingly to improve the mental health of school-age children.

## Background

A consequence of China’s economic reform policy was a great number of rural people moved from rural residence to urban cities to find better jobs and better living conditions. Hukou refers to the Chinese household registration system, defining where a person comes from. Hukou can be categorized into rural hukou and urban hukou. Chinese residents’ access to educational resources, social and healthcare benefits in local areas is bound to their hukou. In China, rural-to-urban migrant children (labeled as “migrant children” henceforth) are those who under 18 and have shifted from rural residence to urban cities for at least 6 months without urban hukou [1]. To date, approximately 35.8 million migrant children have moved from original rural residence to urban areas with their parents [2].

Changes in the living environment could lead to negative effects on migrant children’s mental health [3]. The Strengths and Difficulties Questionnaire (SDQ) is a widely used instrument that assesses children’s mental health across international settings and a higher total difficulties score of SDQ reflects poorer mental health status [4]. The migration process could lead to emotional symptoms, conduct problems, hyperactivity/inattention, peer problems, poorer prosocial behavior and a higher total difficulties score [2]. Urban children are those who were born and live in urban residence. Several studies have compared migrant children’s mental health to their urban counterparts’. For example, two studies with representative samples found that migrant children reported a higher total difficulties score and a higher prevalence of mental health problems than urban youths [2, 5]. However, not all migrant children reported different results compared to urban peers’. For example, a school-based survey indicated that migrant children reported equal mental wellbeing in comparison to urban children [6]. Therefore, mental health status disparities between migrant and urban children remain unclear.

Different from migrant children and urban children, rural children are those who were born and live in rural residence. Left-behind children are those who were left by their parents in the original rural places when their parents moved to urban cities. Previous research has focused on left-behind children’s psychological outcomes, whereas limited attention has been paid to rural children’s mental health and the disparities between other children [7]. As far as we are aware, only two studies with representative samples have compared mental health outcomes between rural children and urban children using the SDQ. The first one was conducted in India and rural children reported more mental health problems [5]. However, due to two schools participated in this study were single-gender schools which might limit the external validity of the study. The second one was conducted involving eight provinces of China. Although the sample size was large in this study, the mental health outcomes reported merely SDQ scores without the prevalence rate of rural and urban children’s mental health problems respectively, which might not be able to provide great insight into rural and urban children’s psychological outcomes [8].

Far less attention has been paid to illustrating the mental health outcomes differences between migrant and rural children. To our knowledge, only one study has found that migrant children reported a lower total difficulties score in comparison to left-behind children [9]. Another study conducted in Moldova reported that left-behind children had more conduct problems but not emotional symptoms than migrant peers [10]. However, left-behind children seemed to be a special group in rural children whose physiological effects might not necessarily represent all the rural children’s.

In addition to migrant status, there were several other socio-demographic characteristics that influenced children’s mental health. Those socio-demographic characteristics included individual characteristics (e.g., age, gender, insurance status), family-related factors (e.g., parental education level, whether living with parents, whether often communicating with parents, family economic status, family climate) and school-related factors (e.g., relationships with others, school achievement). Exploring the relationships between socio-demographic characteristics and children’s mental health outcomes might yield suggestions to relieve their mental health burden. A large body of studies identified that socio-demographic characteristics were associated with children’s mental health, including older age, difficulties in relationships with others, difficulties communicating with parents, not living with parents, and poor school achievement as risk-enhancing factors [5, 9, 11], whereas higher family economic status and higher parental education level, satisfactory family climate as risk-attenuating factors [2, 12, 13].

Although prior research has examined the associations between these factors mentioned above and children’s mental health outcomes empirically, several predictors’ effects remain unclear. For example, the female gender has received mixed support [2, 5, 14]. In addition, the associations between insurance status and children’s mental health status have not been well explored. In the current study, we hypothesized that children insured would have better mental health status since several necessary mental healthcare benefits were covered by insurance plans.

Taken together, there are at least four main research gaps in the existing literature and this study aims to fill. First, rural children’s mental health status remains limited explored. Prior studies paid much attention to left-behind children’s mental health conditions [7, 9, 15]. However, findings regarding left-behind children’s mental wellbeing might not be representative of the whole rural children’s. Second, findings regarding the mental health disparities among three groups of school-age children were less clear after controlling for socio-demographics. Third, not all socio-demographics have been explored among children, insurance status, for example. Fourth, none of the studies have demonstrated the relationships between mental health conditions and socio-demographics among these three groups of school-age children by the same questionnaire at the same time point, since studies conducted using different instruments could draw different conclusions.

The purpose of this study was to compare mental health status among migrant, urban and rural school-age children. As previous studies reported, socio-demographic factors could affect children’s mental health [2, 12, 13]. Therefore, we subsequently compared the psychological outcomes of these three groups of children after controlling for socio-demographics, including individual characteristics, family-related factors, and school-related factors.

## Methods

### Study participants and procedures

We conducted a cross-sectional survey involving rural, migrant and urban children in Guangdong Province, which is one of the most developed provinces in China. The data was collected between October 2014 and February 2015. Guangzhou is the provincial capital and one of the most developed cities in Guangdong Province. In contrast, Meizhou is one of the cities with the lowest Gross Domestic Product in Guangdong Province.

Schools located in Guangzhou included private schools and public schools. However, schools located in Meizhou only included village schools. Stratified cluster sampling of classes was based on 10 districts of Guangzhou, 5 villages of Meizhou, and 3 school types. Due to the lack of urban hukou, migrant children were not able to attend public schools but to attend private schools. Compared to public schools, private schools and village schools had limited teaching facilities and educational resources.

Four districts were selected from 10 districts in Guangzhou by convenience sampling. For the purpose of our study, we selected 3 private primary schools and 4 public primary schools by random sampling in these 4 districts. One class in Grade 5 and one class in Grade 6 were randomly selected in each school as well. As for rural children, first, we selected village 1 and village 2 from 5 villages in Meizhou by convenience sampling. Then, we selected one primary school in villages 1 by random sampling. Next, one class in Grade 5 and one class in Grade 6 in the school were randomly selected. Due to the small population in this school recruited, we tried to survey rural children in the same grade from other primary schools in village 2. However, headmasters of primary schools in village 2 were not willing to participate for lack of time or worried about privacy. Thus, we selected a middle school via convenience sampling in village 2. Finally, one class in Grade 7 and one class in Grade 8 in this middle school were randomly selected.

Students who were absent during the survey were excluded, and those urban children with urban hukou enrolled in private schools were excluded as well. All participants provided their socio-demographic information and completed SDQ. The total assessment took them approximately 25 min to complete. Information from three groups of children was fully-identified or cleaned to ensure the data was eligible. The initial sample consisted of 282 rural children, 395 migrant children and 272 urban children. Of 282 rural children, 14 cases were dropped from this study due to missing data (*n* = 6) or being identified as multiple choices for single-choice items (*n* = 8). Of 395 migrant children, 23 cases were dropped due to missing data (*n* = 8), being identified as multiple choices for single-choice items (*n* = 9) or having moved to Guangzhou less than 6 months (*n* = 6). Among 272 urban children, 18 urban children were dropped due to missing data (*n* = 6), being identified as multiple choices for single-choice items (*n* = 5) or private school attendances (*n* = 7). Finally, we included 268 rural children, 372 migrant children and 254 urban children. The valid response rate of rural, migrant and urban children was 95.0, 94.2 and 93.4%, respectively.

### Measures

The SDQ includes 25 items divided into 5 subscales: Emotional Symptoms, Conduct Problems, Hyperactivity/Inattention, Peer Problems and Prosocial Behaviour. Participants rated items on each subscale from 0 (not true) to 2 (certainly true). The sum of scores from each five items in each subscale generates its score (range 0–10). The prosocial behaviour score is excluded from the total difficulties score since a higher prosocial behaviour score indicates a better mental health condition whereas a higher total difficulties score indicates poorer mental health status [16]. Therefore, the total difficulties score is generated by summing the scores in the first four subscales (range 0–40).

Following the recommendations of Goodman and other prior studies, the cut-off scores for mental health problems evaluations were based on the total difficulties score of SDQ [4, 17, 18]. The SDQ scores above the 90th percentile could raise the possibility of a diagnosed mental health problem [16]. Previous studies showed that the 10% of the highest scores of a sample were classified as abnormal, the next 10% as borderline, and the remaining 80% as normal to identify those who were at high risk [4, 8, 17–21]. Therefore, those children with a total difficulties score ≥ 90th percentile and from 80th to 90th percentile for the highest total difficulties score among 894 participants were categorized into abnormal and borderline group, respectively. In order to examine those children with psychological problems as many as possible, children with a total difficulties score ≥ 80th percentile were defined as having mental health problems in this study. The absolute cut-points for the 90th percentile and 80th percentile were 19 and 15, respectively. The Cronbach’s α for the SDQ in this study was 0.78.

### Socio-demographic characteristics

During our study, participants provided information about their gender, age, insurance status (whether they were insured), father’s education level (primary education or less; or secondary education; or university), mother’s education level (primary education or less; or secondary education; or university), living status (whether they lived with their parents). We also included variables indicating whether participants often communicating with their parents (seldom or often), monthly household income (less than 3000 RMB or more), how did participants feel about family climate (unsatisfactory or satisfactory), whether participants got on well with teachers (yes or not) and participants’ school achievement (good or poor).

### Data analysis

We first used Bonferroni-corrected one-way analyses of variance (ANOVAs) and the Fisher’s exact tests to assess the differences of the variables included in our study between dropped and retained participants in each group. Then, we assessed the distribution of participants’ socio-demographic characteristics in each group. Subsequently, the mean SDQ scores of each group were calculated. We used one-way ANOVAs and Bonferroni post hoc test to evaluate SDQ scores differences between every two groups. In order to have a better understanding of children’s mental health problems, we also used chi-square analyses to assess differences in the prevalence of mental health problems among three groups of children. Next, we ran a multiple linear regression analysis to measure mental health differences among the three groups of children after controlling for socio-demographics. Data analyses were conducted in SPSS 22.0.

## Results

### Missing data analysis

Rural children dropped due to missing data reported significantly higher father’s education level (Fisher’s exact test, *p* = 0.010), significantly more often communicating with parents (Fisher’s exact test, *p* = 0.037), significantly better relationship with teachers (Fisher’s exact test, *p* = 0.013), and significantly lower Hyperactivity/Inattention score (F = 5.679, *p* = 0.018), compared to rural children who were included. Migrant children dropped due to missing data reported significantly older age (Fisher’s exact test, *p* = 0.008), and significantly better school achievement (Fisher’s exact test, *p* = 0.025), compared to migrant children who were included. Urban children dropped due to missing data reported significantly older age (Fisher’s exact test, *p* = 0.001), significantly lower father’s education level (Fisher’s exact test, *p* < 0.001), and significantly lower mother’s education level (Fisher’s exact test, *p* = 0.001), compared to urban children who were included.

### Participants’ socio-demographic characteristics

Table 1 presents the participants’ socio-demographic characteristics. Rural group had more girls and was on average older than the other two groups. Nearly one third (29.5%) of rural children were left-behind children, not living with parents. Rural families had the largest proportion (88.4%) of lower monthly household income (defined as monthly household income less than 3000 RMB). In urban group, 82.3% of children reported being satisfied with the family climate, while the figure for rural children was only 29.9%. Rural children reported the largest percentage (69.4%) of not getting along well with teachers while migrant children had the largest proportion (55.9%) of poor school achievement.

**Table 1:** Socio-demographic characteristics of participants in each group n (%)/M (SD)

Variables	Migrant (*n* = 372)	Urban (*n* = 254)	Rural (*n* = 268)
Gender			
Male	201 (54.0)	137 (53.9)	109 (40.7)
Female	171 (46.0)	117 (46.1)	159 (59.3)
Age	11.52 ± 0.70	11.46 ± 0.57	13.02 ± 2.14
11–13	367 (98.7)	253 (99.6)	185 (69.0)
14–17	5 (1.3)	1 (0.4)	83 (31.0)
Insurance status			
Uninsured	58 (15.6)	3 (1.2)	35 (13.1)
Insured	314 (84.4)	251 (98.8)	233 (86.9)
Father’s education level			
Primary education or less	50 (13.4)	8 (3.1)	43 (16.0)
Secondary education	277 (74.5)	97 (38.2)	177 (66.1)
University	45 (12.1)	149 (58.7)	48 (17.9)
Mother’s education level			
Primary education or less	90 (24.2)	7 (2.8)	92 (34.3)
Secondary education	251 (67.5)	110 (43.3)	169 (63.1)
University	31 (8.3)	137 (53.9)	7 (2.6)
Living with parents			
Yes	353 (94.9)	245 (96.5)	189 (70.5)
No	19 (5.1)	9 (3.5)	79 (29.5)
Communicating with parents			
Often	242 (65.1)	197 (77.6)	148 (55.2)
Seldom	130 (34.9)	57 (22.4)	120 (44.8)
Monthly household income (RMB)			
Less than 3000	257 (69.1)	69 (27.2)	237 (88.4)
No less than 3000	115 (30.9)	185 (72.8)	31 (11.6)
Family climate			
Satisfactory	290 (78.0)	209 (82.3)	80 (29.9)
Unsatisfactory	82 (22.0)	45 (17.7)	188 (70.1)
Getting on well with teachers			
Yes	304 (81.7)	215 (84.6)	82 (30.6)
Not	68 (18.3)	39 (15.4)	186 (69.4)
School achievement			
Good	164 (44.1)	145 (57.1)	119 (44.4)
Poor	208 (55.9)	109 (42.9)	149 (55.6)

*M* Mean, *SD* Standard deviation

### SDQ scores

SDQ scores of migrant, urban and rural school-age children are described in Table 2. Table 3 shows the results of multiple comparisons of SDQ scores of three groups of children by Bonferroni post hoc test. Bonferroni post hoc test showed that migrant children and rural children reported significantly higher scores than urban peers in emotional symptoms, hyperactivity/inattention and total difficulties score (*p* < 0.01). In addition, migrant children reported a higher peer problems score compared to urban children (*p* < 0.001).

**Table 2:** SDQ scores of migrant, urban and rural children (M ± SD)

Subscales	Migrant (*n* = 372)	Urban (*n* = 254)	Rural (*n* = 268)	F	*p*
Emotional Symptoms	2.47 ± 2.15	1.85 ± 2.02	2.82 ± 2.29	13.429	< 0.001***
Conduct Problems	2.16 ± 1.68	2.09 ± 1.61	2.19 ± 1.56	0.288	0.749
Hyperactivity/Inattention	3.26 ± 1.96	2.72 ± 2.11	3.50 ± 2.05	10.043	< 0.001***
Peer Problems	3.69 ± 1.63	3.19 ± 1.54	3.38 ± 1.62	7.770	< 0.001***
Prosocial Behaviour	7.20 ± 1.98	7.35 ± 2.11	7.15 ± 2.05	0.676	0.509
Total Difficulties Score	11.58 ± 5.33	9.85 ± 5.27	11.90 ± 5.26	11.531	< 0.001***

*M* Mean, *SD* Standard deviation, *SDQ* Strengths and Difficulties Questionnaire; *** *p* < 0.001

**Table 3:** Multiple comparisons of SDQ scores of children by Bonferroni post hoc test

						95% Confidence Interval	
Subscales	I	J	Mean Difference (I-J)	Std. Error	*p*	Lower Bound	Upper Bound
Emotional Symptoms	Rural	Urban	0.97	0.19	< 0.010**	0.51	1.42
		Migrant	0.35	0.17	0.131	−0.07	0.76
	Migrant	Urban	0.62	0.18	0.001**	0.20	1.04
Conduct Problems	Rural	Urban	0.10	0.14	1.000	−0.24	0.45
		Migrant	0.03	0.13	1.000	−0.29	0.34
	Migrant	Urban	0.08	0.13	1.000	−0.24	0.40
Hyperactivity/Inattention	Rural	Urban	0.78	0.18	< 0.001***	0.35	1.21
		Migrant	0.25	0.16	0.382	−0.14	0.64
	Migrant	Urban	0.53	0.17	< 0.010**	0.13	0.93
Peer Problems	Rural	Urban	0.20	0.14	0.494	−0.14	0.53
		Migrant	−0.31	0.13	0.052	−0.61	0.00
	Migrant	Urban	0.50	0.13	< 0.001***	0.19	0.82
Prosocial Behaviour	Rural	Urban	−0.20	0.18	0.779	−0.63	0.23
		Migrant	−0.06	0.16	1.000	−0.45	0.33
	Migrant	Urban	−0.14	0.17	1.000	−0.54	0.26
Total Difficulties Score	Rural	Urban	2.05	0.46	< 0.001***	0.93	3.16
		Migrant	0.32	0.42	1.000	−0.70	1.33
	Migrant	Urban	1.73	0.43	< 0.001***	0.70	2.76

*SDQ* Strengths and Difficulties Questionnaire; ** *p* < 0.01, *** *p* < 0.001

### Total difficulties score among three groups of school-age children after controlling for socio-demographics

Table 4 shows the result of total difficulties score among three groups of school-age children after controlling for socio-demographics. In multiple linear regression analysis, we found rural children and migrant children reported significantly a higher total difficulties score than urban children (*p* = 0.046 and 0.024, respectively). Additionally, we found female gender, having insurance, seldom communicating with parents, and higher monthly household income (defined as no less than 3000 RMB) were negatively associated with a higher total difficulties score. Conversely, children’s father with secondary education was positively associated with a higher total difficulties score.

**Table 4:** Total difficulties score among three groups of school-age children after controlling for socio-demographics

Covariates	Unstandardized coefficients		*p*
	β	Std. Error	
Constant	14.164	1.095	< 0.001***
Groups of children (vs. Urban children)			
Migrant children	1.235	0.545	0.024*
Rural children	1.428	0.716	0.046*
Gender (vs. Male)			
Female	−1.009	0.347	0.004**
Age (vs.11–13)			
14–17	−0.280	0.643	0.664
Insurance status (vs. Uninsured)			
Insured	−3.618	0.661	< 0.001***
Father’s education level (vs. Primary education or less)			
Secondary education	1.628	0.735	0.027*
University	1.406	1.027	0.171
Mother’s education level (vs. Primary education or less)			
Secondary education	−0.982	0.607	0.106
University	1.216	1.062	0.253
Living with parents (vs. Yes)			
No	−1.042	0.689	0.131
Communicating with parents (vs. Often)			
Seldom	−1.753	0.472	< 0.001***
Monthly household income (RMB) (vs. Less than 3000)			
No less than 3000	−2.399	0.472	< 0.001***
Family climate (vs. Satisfactory)			
Unsatisfactory	1.002	0.510	0.050
Getting on well with teachers (vs. Yes)			
Not	−0.008	0.528	0.988
School achievement (vs. Good)			
Poor	0.120	0.404	0.767

*p < 0.05, **p < 0.01, ***p < 0.001

### Prevalence of mental health problems

As mentioned above, children with the total difficulties score of SDQ in the borderline or abnormal category were defined as having mental health problems. The prevalence rate (not shown) of mental health problems among rural, migrant and urban children was 26.5, 18.8 and 15.0% (χ^2^ = 11.41, *p* = 0.003), respectively. Rural children reported a significantly higher prevalence rate of mental health problems than urban children (χ^2^ = 10.50, *p* < 0.001), which was within expectations. The comparisons of the prevalence rate of mental health problems between rural and migrant children and between migrant and urban children did not reach statistical significance.

## Discussion

We compared SDQ scores and the prevalence rate of mental health problems among three groups’ school-age children in Guangdong Province, China. The study yielded several key findings. First, migrant children and rural children reported significantly higher scores than urban peers in emotional symptoms, hyperactivity/inattention and total difficulties score. In addition, migrant children reported a higher peer problems score compared to urban children. Second, after controlling for socio-demographics, we found rural children and migrant children reported significantly a higher total difficulties score than urban children, indicating that rural children and migrant children had poorer mental health than urban children. Third, rural children reported significantly a higher prevalence rate of mental health problems than urban children. Fourth, we found female gender, having insurance, seldom communicating with parents, and higher monthly household income were negatively associated with a higher total difficulties score. Taken together, the findings provided additional evidence that the mental health status of school-age children with different backgrounds.

This is the first study to our knowledge to compare mental health status among three groups’ school-age children at the same time point. As such, we are not able to compare to any other study that documents the mental health status of three groups’ children in one context. In comparison to representative samples in previous findings, we found that left-behind children in rural areas reported a higher total difficulties score and a higher prevalence rate of mental health problems than urban children which could be applied to rural and urban children in this case [5]. In the present study, rural children reported a smaller proportion of higher monthly household income compared to urban children (11.6% vs. 72.8%). In multiple linear regression analysis, we also found higher monthly household income was negatively associated with a higher total difficulties score. This result suggested that higher monthly household income might serve as a protective factor for children’s mental health. Therefore, the disparities between rural and urban children might be a result of family economic status disparities, which was consistent with previous studies [8, 15, 22, 23].

The findings regarding the total difficulties score and the prevalence of mental health problems between migrant children and urban children aligned with those of prior studies as well, which indicated migrant children suffered more mental health problems than urban children [2, 5]. One possible explanation was that, as mentioned above, migrant children had less access to several medical resources in urban areas due to lack of urban hukou, since several healthcare benefits were just limited to urban children. Migrant children were not able to receive psychological support from healthcare institutions when they had a psychological burden, which might jeopardize migrant children’s mental health [2]. Alternatively, in this study, migrant children had the lowest proportion of having insurance. In the multiple linear regression analysis, we also found that having insurance was negatively associated with a higher total difficulties score. Those children who were not insured needed to pay more if they wanted to receive several necessary health services due to their uninsured status. It was possible that a number of uninsured migrant children might not want to receive such medical support, which in turn harmed their mental health.

The significant association between female gender and total difficulties score among children was consistent with a prior study showing that female gender was associated with better mental health status [5]. Often communicating with parents was associated with a higher total difficulties score, which was inconsistent with other prior research conducted among children [15, 24]. One possibility was the differences in their communication context. For example, if parent-children communication related to life difficulties, children’s mental health would be even worse. However, if the parent-children communication was more involved in children’s feelings or academics, children’s psychological burdens would be relieved [25].

Previous study suggested that higher parental education level would mitigate against the negative migration effects on children. Thus, higher parental education level served as a protector in children’s mental wellbeing [2]. However, in multiple linear regression analysis, we found children’s father with secondary education was positively associated with a higher total difficulties score. One explanation would be that children’s father with primary education or less was more likely to be unemployed. Thus, they could have more time to interact with children or care about their children’s psychological development, giving children emotional support when necessary.

## Limitations and strengths

This study had several limitations. First, we assessed children’s mental health status by self-report questionnaire, other measures that took into account the assessment of children’s mental health from teachers’ and parents’ reports would provide more precise estimates. Second, some constructs (e.g., family climate, communicating with parents) were brief but not thorough, based on several questions. The limited item sets might limit the reliability and validity of the assessment of these predictors. Third, this study was a cross-sectional study. Although we examined several socio-demographics related to children’s mental health status, we could not found the causal direction. Finally, there were systematic differences of these socio-demographics included in our study between dropped and retained participants in each group, which might limit the external validity of this study.

Despite these limitations, especially given the large sample size, this is the first study that represents a significant step in understanding mental health conditions among school-age children with a variety of backgrounds in one context. The results add to the existing literature on how individual characteristics, family-related factors and school-related factors influence three groups of school-age children’s mental health. A further understanding of this issue will provide insight into some of the variability in psychological functioning among school-age children and have significant implications for mental health services provided for school-age children, especially rural and migrant school-age children, in order to improve their mental health conditions.

## Conclusions

The present study documented mental health status among migrant, urban and rural children in Guangdong Province, China. Rural children and migrant children reported poorer mental health than urban children. Female gender, having insurance, seldom communicating with parents, and higher monthly household income were associated with better mental health of children. However, children’s father with secondary education was associated with poorer mental health of children. Given the different effects of socio-demographics, further support might be provided accordingly in order to improve the mental health of school-age children.

## Data Availability

The datasets used and analyzed during the current study are available from the corresponding author on reasonable request.

## Abbreviations

ANOVAs: Analyses of variance
M: Mean
SD: Standard deviation
SDQ: Strengths and Difficulties Questionnaire

## References

[1] Liang Z, Guo L, Duan C (2008). Migration and the well-being of children in China. Yale-China Health J.

[2] Lu J, Wang F, Chai P, Wang D, Li L, Zhou X (2018). Mental health status, and suicidal thoughts and behaviors of migrant children in eastern coastal China in comparison to urban children: a cross-sectional survey. Child Adolesc Psychiatry Ment Health.

[3] Kim J, Nicodimos S, Kushner SE, Rhew IC, McCauley E, Vander SA (2018). Comparing mental health of US children of immigrants and non-immigrants in 4 racial/ethnic groups. J Sch Health.

[4] Goodman R (1997). The strengths and difficulties questionnaire: a research note. J Child Psychol Psychiatry.

[5] Nair S, Ganjiwale J, Kharod N, Varma J, Nimbalkar SM (2017). Epidemiological survey of mental health in adolescent school children of Gujarat, India. BMJ Paediatr Open.

[6] Cheung NW (2013). Rural-to-urban migrant adolescents in Guangzhou, China: psychological health, victimization, and local and trans-local ties. Soc Sci Med.

[7] Huang GW, Du QY, Liu ZY, Liu YP, Huang Q, Wu H (2010). Analysis on influences of guardians on behavior of left-behind children aged 3 to 7 years in countryside. Zhonghua Er Ke Za Zhi.

[8] Gao X, Shi W, Zhai Y, He L, Shi X (2013). Results of the parent-rated strengths and difficulties questionnaire in 22108 primary school students from 8 provinces of China. Shanghai Arch Psychiatry.

[9] Wang F, Zhou X, Hesketh T (2017). Psychological adjustment and behaviours in children of migrant workers in China. Child Care Health Dev.

[10] Vanore M, Mazzucato V, Siegel M (2015). ‘Left behind’ but not left alone: parental migration & the psychosocial health of children in Moldova. Soc Sci Med.

[11] Lau W, Silove D, Edwards B, Forbes D, Bryant R, McFarlane A (2018). Adjustment of refugee children and adolescents in Australia: outcomes from wave three of the building a new life in Australia study. BMC Med.

[12] Lesinskiene S, Girdzijauskiene S, Gintiliene G, Butkiene D, Puras D, Goodman R (2018). Epidemiological study of child and adolescent psychiatric disorders in Lithuania. BMC Public Health.

[13] Wille N, Bettge S, Ravens-Sieberer U (2008). Risk and protective factors for children's and adolescents' mental health: results of the BELLA study. Eur Child Adolesc Psychiatry.

[14] Fausiah F, Turnip SS, Hauff E (2019). Community violence exposure and determinants of adolescent mental health: a school-based study of a post-conflict area in Indonesia. Asian J Psychiatr.

[15] Wang F, Lin L, Xu M, Li L, Lu J, Zhou X (2019). Mental health among left-behind children in rural China in relation to parent-child communication. Int J Environ Res Public Health.

[16] Goodman R (2001). Psychometric properties of the strengths and difficulties questionnaire. J Am Acad Child Adolesc Psychiatry.

[17] Anmyr L, Larsson K, Olsson M, Freijd A (2012). Strengths and difficulties in children with cochlear implants-comparing self-reports with reports from parents and teachers. Int J Pediatr Otorhinolaryngol.

[18] Goodman R (2015). Youth in mind. SDQ: information for researchers and professionals about the Strengths and Difficulties Questionnaires.

[19] Bourdon KH, Goodman R, Rae DS, Simpson G, Koretz DS (2005). The strengths and difficulties questionnaire: U.S. normative data and psychometric properties. J Am Acad Child Adolesc Psychiatry.

[20] Smedje H, Broman JE, Hetta J, von Knorring AL (1999). Psychometric properties of a Swedish version of the “strengths and difficulties questionnaire”. Eur Child Adolesc Psychiatry.

[21] Van Roy B, Groholt B, Heyerdahl S, Clench-Aas J (2006). Self-reported strengths and difficulties in a large Norwegian population 10-19 years: age and gender specific results of the extended SDQ-questionnaire. Eur Child Adolesc Psychiatry..

[22] Hourigan SE, Southam-Gerow MA, Quinoy AM (2015). Emotional and behavior problems in an urban pediatric primary care setting. Child Psychiatry Hum Dev.

[23] Feinberg ME, Button TM, Neiderhiser JM, Reiss D, Hetherington EM (2007). Parenting and adolescent antisocial behavior and depression: evidence of genotype x parenting environment interaction. Arch Gen Psychiatry.

[24] Yu S, Clemens R, Yang H, Li X, Stanton B, Deveaux L (2006). Youth and parental perceptions of parental monitoring and parent-adolescent communication, youth depression, and youth risk behaviors. Soc Behav Pers.

[25] Guang Y, Feng Z, Yang G, Yang Y, Wang L, Dai Q (2017). Depressive symptoms and negative life events: what psycho-social factors protect or harm left-behind children in China?. BMC Psychiatry.

